# A deep intronic *PHEX* variant associated with X-linked hypophosphatemia in a Finnish family

**DOI:** 10.1093/jbmrpl/ziae169

**Published:** 2024-12-23

**Authors:** Laura Koponen, Minna Pekkinen, Jelmer Legebeke, Mari Muurinen, Salla Rusanen, Shabir Hussain, Fan Wang, Pasi I Nevalainen, Outi Mäkitie

**Affiliations:** Research Program for Clinical and Molecular Metabolism, Faculty of Medicine, University of Helsinki, Helsinki 00014, Finland; Folkhälsan Research Center, Helsinki 00290, Finland; Research Program for Clinical and Molecular Metabolism, Faculty of Medicine, University of Helsinki, Helsinki 00014, Finland; Folkhälsan Research Center, Helsinki 00290, Finland; Children’s Hospital, University of Helsinki and Helsinki University Hospital, Helsinki 00014, Finland; Department of Molecular Medicine and Surgery, Karolinska Institutet, Stockholm 17177, Sweden; Research Program for Clinical and Molecular Metabolism, Faculty of Medicine, University of Helsinki, Helsinki 00014, Finland; Folkhälsan Research Center, Helsinki 00290, Finland; Children’s Hospital, University of Helsinki and Helsinki University Hospital, Helsinki 00014, Finland; Folkhälsan Research Center, Helsinki 00290, Finland; Research Program for Clinical and Molecular Metabolism, Faculty of Medicine, University of Helsinki, Helsinki 00014, Finland; Department of Molecular Medicine and Surgery, Karolinska Institutet, Stockholm 17177, Sweden; Rare Diseases Unit and Endocrine Unit, Department of Internal Medicine, Tampere University Hospital, and Faculty of Medicine and Health Technology, Tampere University, Tampere 33101, Finland; Research Program for Clinical and Molecular Metabolism, Faculty of Medicine, University of Helsinki, Helsinki 00014, Finland; Folkhälsan Research Center, Helsinki 00290, Finland; Children’s Hospital, University of Helsinki and Helsinki University Hospital, Helsinki 00014, Finland; Department of Molecular Medicine and Surgery, Karolinska Institutet, Stockholm 17177, Sweden; Clinical Genetics, Karolinska University Hospital, Stockholm 17177, Sweden

**Keywords:** XLH, genetic skeletal disorders, splicing, next generation sequencing (NGS), whole genome sequencing, transcriptomics

## Abstract

Hypophosphatemic rickets is a rare bone disease characterized by short stature, bone deformities, impaired bone mineralization, and dental problems. Most commonly, hypophosphatemic rickets is caused by pathogenic variants in the X-chromosomal *PHEX* gene, but autosomal dominant and recessive forms also exist. We investigated a Finnish family in which the son (index, 29 yr) and mother (56 yr) had hypophosphatemia since childhood. Both patients had typical clinical, radiographic, and biochemical features of hypophosphatemic rickets, including a pathological fracture in the son. Gene panels and whole-exome sequencing did not reveal any pathogenic variants in the known hypophosphatemia genes. Therefore, we performed whole genome sequencing and identified a deep intronic variant (c.2147 + 1197A > G) in *PHEX*. Both the affected individuals, but none of the unaffected family members, had the same variant, as confirmed by Sanger sequencing. According to RT-PCR, whole transcriptomic data, and in silico analyses, the variant led to a new splice donor site in intron 21 and an 84 basepair pseudoexon between exons 21 and 22, likely leading to the synthesis of abnormal PHEX protein. Our study underscores the importance of intronic *PHEX* variants in X-linked hypophosphatemia (XLH). In patients with features of XLH but negative gene panel or whole-exome sequencing results, the combination of whole-genome sequencing and whole transcriptomics should be considered to detect possible deep intronic variants. The methodologies presented have the potential to be used more widely in other rare diseases.

## Introduction

X-linked hypophosphatemia (XLH, MIM #307800) is a rare inherited skeletal disorder caused by mutations in *PHEX* (phosphate-regulating gene with homologies to endopeptidases on the X chromosome, MIM *300550)*;* the inheritance of XLH follows an X-linked dominant pattern. Characteristic features include low serum phosphate level due to excessive phosphate excretion in urine, leading to rickets and impaired longitudinal growth, bone deformities, fractures, dental abscesses, bone pain, and muscle weakness.^1^^,^^2^

PHEX is a zinc-dependent protease, expressed in osteoblast lineage cells, and recent studies have shown that the zinc-binding site may play a significant role in regulating FGF23.^3^^,^^4^ Other inherited forms of hypophosphatemia are also known, such as autosomal dominant hypophosphatemic rickets due to *FGF23* variants (ADHR, MIM #193100), autosomal recessive hypophosphatemic rickets due to *DMP1* (dentin matrix protein 1) (ARHR1, MIM #241520) or *ENPP1* variants (ectonucleotide pyrophosphatase/phosphodiesterase 1) (ARHR2, MIM #613312), and the extremely rare Raine syndrome (RNS, MIM #259775) due to *FAM20C* variants (Golgi associated secretory pathway kinase).^1^

More than 870 variants of the *PHEX* gene have been identified to cause X-linked hypophosphatemia, most of which are exonic single-nucleotide variants (37%), splicing variants (22%), or small deletions (19%).^5^ Two deep intronic variants were reported in the *PHEX* database (https://www.rarediseasegenes.com/). A variant in intron 7 (c.849 + 1268G > T) leads to the creation of 3 pseudoexons, a premature stop codon, and disruption of PHEX function.^6^ Grimbly et al. have recently reported another deep intronic variant (c.2147 + 1197A > G) in intron 21, causing a premature stop codon, and an 84 basepairs (bp) pseudoexon between exons 21 and 22, predicted to lead to changes in protein function.^7^

Conventional XLH treatment includes oral phosphate to compensate for urinary phosphate loss and active vitamin D to normalize 1,25-OH_2_-vitamin D concentration, improve phosphate absorption and prevent hyperparathyroidism.^8^ Burosumab, an anti-FGF23 monoclonal antibody, is a novel targeted therapy for XLH but is not effective for genetic hypophosphatemias with normal FGF23.^9–11^ Accurate genetic diagnosis is important for appropriate treatment and genetic counseling for patients with hypophosphatemia. Recent advances in DNA- and RNA-based tools have increased the likelihood of successful genetic diagnostics.^7^^,^^12^^,^^13^

In this study, we examined a young adult male and his mother who both had typical clinical and biochemical features of XLH since childhood; however genetic studies showed negative results in *PHEX* sequencing and repeated gene panel studies. Whole exome sequencing (WES) failed to detect the disease-causing deep intronic *PHEX* variant, which was detected only by whole genome sequencing (WGS). The variant has previously been reported in one family but using different methodololgy.^7^ RT-PCR and transcriptomic analysis on patient fibroblasts suggested that the variant leads to an 84 bp pseudoexon between exons 21 and 22.

## Materials and methods

### Study participants

This study was approved by the Research Ethics Committee of the Helsinki University Hospital (HUS/265/2023), and all parts of the study were performed according to the ethical principles defined in the Declaration of Helsinki. All participants signed a written informed consent before participation.

We investigated a Finnish family in which the son (index, 29 yr) and mother (56 yr) had hypophosphatemia since childhood. Both presented with hypophosphatemic rickets, short stature, and varus deformity of the legs, and the index patient had dental abscesses. They had received treatment with oral phosphate and active vitamin D. Despite earlier genetic studies (karyotyping, *PHEX* Sanger sequencing and hypophosphatemia gene panels), the disease-causing gene remained unknown. Therefore, we invited the 2 affected individuals and their unaffected family members to the study, including the index patient’s son, sister, father, and mother’s sister.

For transcriptome analysis, we obtained skin biopsies for fibroblast cultures from both affected individuals and from 6 healthy controls.

### DNA extraction

DNA was isolated from whole blood samples using a GeneAid DNA Isolation Kit (Geneaid Biotech Ltd) or from saliva samples using an Oragene DNA Collection Kit (DNA Genotek).

### Next generation sequencing

The WES data for the 4 family samples were obtained at Blueprint Genetics (Espoo, Finland). Samples were prepared and fragmented for sequencing using adapters and a bead-based selection method on an Illumina platform. The data were mirrored against the reference genome GRCh37 (hg19). After generating the WES data, copy number variants (CNVs) were analyzed against 2 different databases (Clinical Genomics Database and Developmental Disorders Genotype–Phenotype Database) containing more than 3700 genes.

Before WES analysis, the data were annotated using Annovar.^14^ Single nucleotide variants (SNVs) were analyzed using the VarAFT software 2.16 (http://varaft.eu). The following criteria were used to filter the variants: (1) the variant occurs with index and mother (affected), but not in other family members; (2) the variant was in the coding (exons) or splicing region; (3) minor allele frequency in different databases (gnomAD v4.0.0, Sisu, VarSome, dbSNP) was <1 %. The American College of Medical Genetics and Genomics (ACMG) criteria were used to classify the variants as benign, likely benign, uncertain, likely pathogenic, or pathogenic, and 6 categories of splicing variants: PVS1, PS3, PP3, BS3, BP4, and BP7.^15^^,^^16^ Variant effects were predicted by different in silico tools, such as SIFT/Provean,^17^^,^^18^ The Combined Annotation Dependent Depletion (CADD) score,^19^ and an automatic mutant analysis tool HOPE.^20^ The variants were visualized by the Integrative Genome Viewer (IGV). The following databases were used to review gene function: Google Scholar (gene name + bone/phosphate/hypophosphatemia) (https://scholar.google.com/), PubMed (gene name + bone/phosphate/hypophosphatemia) (https://pubmed.ncbi.nlm.nih.gov/), Musculoskeletal KP (https://msk.hugeamp.org/), and GWAS Catalog databases (https://www.ebi.ac.uk/gwas/).

We also analyzed CNVs from WES data using 4 different programs with the manufacturer’s default settings: Copy Number Inference From Exome Reads (CoNIFER) v0.2.2.6, eXome-Hidden Markov Model (XHMM) v1.1.7, ExomeDepth v1.1.15, and COpy number Detection by EXome sequencing (CODEX) v1.18.0.

Whole-genome sequencing (WGS) was performed using the Illumina NovaSeq 6000, PCR-free library preparation at Clinical Genomics, Science for Life Laboratory in Stockholm, Sweden. The paired-end (2 × 150-basepair reads) WGS had 30x coverage for the samples, and the reference genome was hg19. The variants were identified using GATK haplotype caller, Manta, Tiddit, CNVnator and ExpansionHunter and ranked based on disease-causing potential. The variants were ranked using the GenMod tool (https://github.com/Clinical-Genomics/genmod), which scores variants based on different parameters, such as population frequency in GnomAD v4.0.0, CADD score, and splice predictions. The top-ranked variants were visualized using the IGV tool on the Scout platform (Clinical Genomics), and we focused on variants in genes linked to hypophosphatemia. The WGS data were also separately analyzed as described in Figure S1. Potential disease-causing variants from WES and WGS analysis were confirmed by Sanger sequencing.

### Fibroblast cultures

Skin fibroblasts from the 2 affected and 2 control individuals were used for RNA extraction.

The remaining 4 healthy controls for transcriptome analyses had been cultured earlier. Fibroblasts were cultured according to a previously reported protocol.^21^ Briefly, fibroblasts were cultured in DMEM (Dulbecco’s Modified Eagle Medium with 4.5 g/L glucose without L-glutamine, Lonza Walkersville, MD USA), 10 or 20 % FBS/FCS, 2 mM (1X) L-alanyl- L-Glutamine, (Gibco, Thermo Fisher Scientific), 50 IU/mL penicillin and 50 μg/mL streptomycin (Gibco, Life Technologies Europe) at 37 C and 5 % CO_2_ on T-75 flasks. The medium was changed every 3-4 d. The cells were cultured to passage 6 and stored in liquid nitrogen.

For RT-PCR analysis, fibroblasts were cultured in T-75 flasks before pelleting cells for RNA extraction. For whole transcriptome analyses, fibroblasts at passage 6 were transferred to 24 well plates (600 000 cells/well). Cells were synchronized to the synthetic phase (S1) and pelleted for RNA extraction. In synchronization, the serum-free medium was changed after 24 hours of transfer to the plates, the serum-containing medium was replaced 24 hours later, and finally, S1 samples were collected after 20 hours.

### RNA extraction

RNA was isolated from fibroblasts using the RNeasy MiniKit (Qiagen) and from whole blood using a PaxGene Blood RNA Kit 50 (Qiagen). RNA extractions were cleaned using Ambion DNA-free kit (Thermo Fisher Scientific). The quality of the samples (RIN scores) was measured using TapeStation 4200 analysis at the Functional Genomics Unit, Biomedicum, Helsinki, Finland.

### RT-PCR and Sanger sequencing

A pseudoexon between exon 21 and 22 in *PHEX* was confirmed by RT-PCR and Sanger sequencing. cDNA was synthesized from the RNA of patient-derived and control fibroblasts using a High-Capacity cDNA Reverse Transcription Kit with RNase Inhibitor (Applied Biosystems by ThermoFisher Scientific, #4374966). PCR and Sanger sequencing were performed using standard methods. The RT-PCR cDNA primers for the amplified pseudoexon are available upon request.

### Whole transcriptome analysis

In total, the whole transcriptome sequencing was performed on 33 RNA samples, which were isolated from the patients’ (*n* = 2) and controls’ (*n* = 6) fibroblasts or the patients’ (*n* = 2) and controls’ (*n* = 2) whole blood. Each sample had 3 replicates from fibroblasts and 2 replicates from whole blood.

RNA library ribo-depletion construction and sequencing (at least 76 M reads per sample) was performed using the FIMM Genomics Next Generation Sequencing Sequencing unit at the University of Helsinki, supported by HiLIFE and Biocenter, Finland. Library preparation and quality control was performed according to the RNA prep Reference Guide (Illumina) and the LabChip GX Touch HT High Sensitivity assay (PerkinElmer), respectively. The pooled libraries were quantified using Collibri Library Quantification kit (Thermo Fisher Scientific). Paired-end sequencing (read length for run was 2 × 151 bp) was performed on the Illumina NovaSeq6000 system using the S4 flow cell (Illumina).

Quality control (QC) of the raw RNA-seq data was performed with FastQC (v0.11.9),^22^ and library adapters were removed with cutadapt (v4.0). Sequence reads were aligned with STAR basic two-pass mode (v2.7.9a)^23^ using human GRCh build 38^24^ and GENCODE v43 gene annotation,^25^ the outFilterScoreMinOverLread and outFilterMatchNminOverLread settings were set to 0.33. The BAM files were subsequently sorted and indexed with SamTools (v1.17).^26^ QC of the BAM files was performed with RSeQC junction annotation and junction saturation (v2.6.4),^27^ Picard insert size, RnaSeqMetrics assignment, RnaSeqMetrics strand mapping, and gene coverage (v2.27.5).^28^ MultiQC (v1.12) was used to combine and assess the quality of the individual output files.^29^

The SpliceAI lookup website (https://spliceailookup.broadinstitute.org/),^30^ NetGene2 (v2.42),^31^^,^^32^ MaxEntScan score splice sites,^33^ and SPiP (v2.1)^34^ were used to predict whether the previously identified deep intronic NM_000444:c.2147 + 1197A > G variant leads to altered RNA splicing. In addition, branch points within the intron were predicted with BPP.^35^ The default settings were used for all the prediction tools. Potential predicted altered RNA splicing events were manually assessed with the IGV (v2.16.0)^36^ using merged participant BAM files comprising the 3 individual BAM files.

## Results

### Clinical and biochemical findings

#### Index

**Figure 1:**
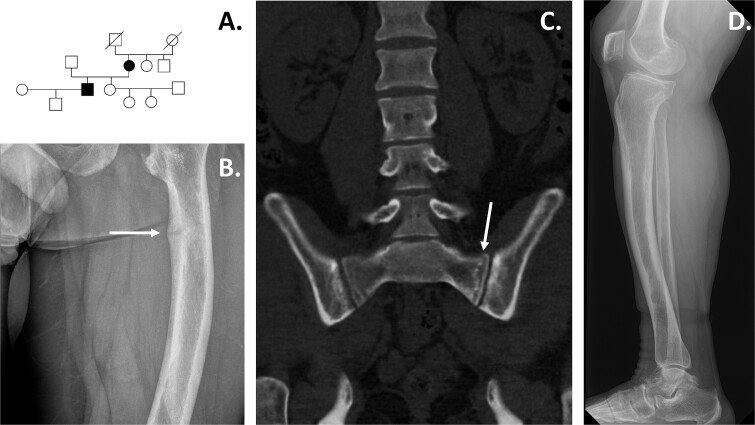
(A) The pedigree of the family with hypophosphatemia. Index and mother are affected (color: black), and other family members are healthy. (B) Radiograph of the index patient’s left femur. The medial diaphyseal cortex displays cortical thickening, along with a distinct bright line indicative of a healing stress fracture with partial reossification. (C) CT image of the index patient’s pelvis. Fracture fissure is running through the lateral right wing of the sacrum, showing some degree of reossification. Conversely, on the left anterolateral sacral wing, there is a well-healed fracture. (D) The lower-leg X-ray of the mother exhibits anterior bowing of the left tibia. Additionally, there is evidence of coarse trabeculation and localized thinning of the endosteal cortex.

The index subject, presently 29 yr old, was born at gestational week 39 + 4 after a normal pregnancy without any health concerns (Figure 1A). Additional laboratory tests were performed because the mother had been diagnosed with hypophosphatemia. At 3 mo, the serum phosphate level was low 1.25 mmol/L. Cholecalciferol supplementation was administered according to general recommendations. At 4 mo of age, he measured 63.3 cm (−1.3 SD) and 7820 g (+20 %) and had pectus excavatum. A wrist X-ray showed slight rachitic changes and a month later he was clearly hypophosphatemic (S-Pi 0.97 mmol/L). Fractional tubular reabsorption of phosphate (TRP) and the ratio of tubular maximum reabsorption of phosphate to glomerular filtration rate (TmP /GFR) indicated phosphate wasting (Table 1). Oral phosphate and alphacalcidol were started. With treatment his pectus excavatum normalized but there was gradual bowing of the legs during follow-up. Knee gap was maximally 11 cm and a proximal tibial osteotomy was performed at the age of 3 yr 3 mo. His adult height is 162 cm (−2.5 SD). He has had repeated dental abscesses. He developed secondary hyperparathyroidism, and 3 parathyroid glands were removed with transient postoperative hypoparathyroidism at the age of 23 yr. He had slight parenchymal calcification of the kidneys, as assessed by computed tomography at the age of 25 yr. His phosphate and alphacalcidol treatments were transiently discontinued (Table 1). However, he developed stress fractures of the left femur and the right sacral area (Figure 1B and C). After restarting the phosphate and alphacalcidol treatments the fractures showed slow healing. The patient had repeatedly high hemoglobin (195 g/L; reference: 134–167 g/L) values since the age of 8 yr; however, the iron status was normal and other investigations showed no abnormalities.

**Table 1:** Biochemical profile of the patients during adulthood. The minimum and maximum values during follow-up are presented. The index was without treatment for 9 mo prior to the “untreated” laboratory tests.

	**Index treated**	**Index untreated**	**Mother treated**	**Mother untreated**	**Reference range**
**Phosphate (mmol/L)**	0.32-1.08^a^	0.48	0.39-0.61	0.33-0.70	0.71-1.41
**Ca++ (mmol/L)**	1.25-1.33^b^	ND	1.23-1.35	1.18-1.34	1.20-1.35
**PTH (pmol/L)**	3.6-6.6^b^	6.6	5.1-10.3	4.1-6.6	1.6-6.9
**Alkaline phosphatase (U/L)**	57-178^c^	172	50-106	59-130	35-105
**FGF23 (kRU/L)**	107	ND	53.5	ND	26-110
**25-OH vitamin D (nmol/L)**	54-108	71	49-106	71-106	50-120
**1,25-OH vitamin D (pmol/L)**	125	ND	56-66	ND	50-215
**TRP (%)**	83	ND	65	ND	85-95
**TmP/GFR (mmol/L)**	0.87	ND	0.42	ND	1.02-2.00
**Urine calcium/ creatinine (mmol/mmol)**	0.21	0.47	3.8	ND	<1.1
**Hemoglobin (g/L), smoking**	164-202	195			134-167
**Hemoglobin (g/L), not smoking**	188-192^d^				134-167
**Hemoglobin (g/L), smoking**			149-173	144-166	117-155
**Hemoglobin (g/L), not smoking**			166^d^	ND	117-155

a phosphate 1500-2750 mg daily divided to 3-5 doses

b before the onset of secondary hyperparathyroidism

c before and after the onset of secondary hyperparathyroidism

d the mother not smoking for 2 wk and the son for 6 mo

Abbreviations: ND, not done; TmP/GFR, the ratio of tubular maximum reabsorption of phosphate to glomerular filtration rate; TRP, fractional tubular reabsorption of phosphate.

#### Mother

The index subject’s mother, now 56 yr old, had slow motor development in early childhood and started walking at the age of 1.5 yr. Soon thereafter she developed a significant genu varum deformity with lower limb pain. Radiographs showed typical changes of rickets in the knees, ankles, and wrists, and bowing of the left tibial bone (Figure 1D). Serum phosphate was low, 0.9 mmol/L, calcium was normal, and alkaline phosphatase (ALP), iron concentrations and hemoglobin were elevated. TRP and TmP/GFR indicated phosphate wasting (Table 1). Vitamin D treatment was used but there was no improvement after 6 mo of treatment. She was given a single vitamin D dose of 250 000 IU orally followed by cholecalciferol drops at 30 μg per day. At the age of 3 yr her serum phosphate was 0.9 mmol/L (reference 21.2-1.8 mmol/L) despite phosphate and cholecalciferol supplementation and later cholecalciferol was switched to dihydrotachysterol. The knee gap was maximally 7 cm but with treatment it almost normalized by the age of 13 yr despite persistent hypophosphatemia. Her adult height is 146 cm (−3.0 SD). As a young adult she had no major clinical problems, and she was treated with alphacalcidol monotherapy. The traumatic fracture of the right IV metatarsal healed slowly. She later presented with musculo-skeletal pain and was found to have calcification of the right rotator cuff and the proximal end of the left clavicle, as well as the right proximal metatarsophalangeal joint. Diurnal urine calcium was 9.6 mmol (reference 21.3–6.5 mmol). Alphacalcidol treatment was discontinued. At 55 yr of age, she started to have nonspecific bone pains and a low dose phosphate therapy was restarted at 250 mg twice daily. She has not sustained any low-energy fractures. The hemoglobin levels remained within or slightly above the reference range and additional investigations did not identify abnormalities in the iron balance.

### Genetic and transcriptomic findings

#### Previous genetic tests

During his clinical follow-up, the index underwent several genetic studies over the years. Karyotype was normal (46, XY). Genome-wide analysis for copy number variations showed a duplication (dup(12)(p13.31p13.31), chr12:g.6309579_6650802dup) but since it did not segregate with hypophosphatemia, it was not considered a likely cause of the disease. The gene panel results were negative for several hypophosphatemia-related genes *OCRL, ALPL, CLCN5, CYP27B1, CYP2R1, DMP1, ENPP1, FAH, FGF23, KL, PHEX, SLC34A1, SLC34A3,* and *VDR* genes (Blueprint Genetics, Finland and Center for Genomics and Transcriptomics, Germany). The single gene sequencing of the *PHEX* and *FGF23* exons was also negative, and no *PHEX* deletions or duplications were found in high-density targeted arrays (Centogene).

#### Exome sequencing

We performed whole exome sequencing of the family. Data analysis excluded again variants in the known hypophosphatemia genes. Search for novel candidate genes identified a rare heterozygous missense variant in *PIK3C2B* (NM_002646: c.875G > A, p.Arg292His) (rs758617979) which was identified in both affected but in none of the 4 unaffected individuals. The CADD score for the variant was 17.15, and the frequencies in the population databases were 0.000008 (dbSNP) and 0.000004020 (GnomAD v.4.0.0). In silico predictions were variable. Varsome classified the variant as benign; however, Provean classified the variant as pathogenic and SIFT as a neutral variant. Based on HOPE predictions, there is a difference in charge between the wild-type and mutant, and this can cause a loss of interactions with other residues. According to the Reactome Pathways tool (www.reactome.org), FGFR binds to PI3K, and this pathway is linked to FGFR and PI3K, indicating that the PI3K pathway may be associated with FGFR and possible hypophosphatemia. Hypophosphatemia has also been reported as a side effect of PI3K inhibitors.^37^ However, according to our knowledge, *PIK3C2B* has not been previously linked to hypophosphatemia, and we regarded the variant as an unlikely cause for the severe hypophosphatemia in our family. We also found 6 other rare variants, but the Combined Annotation Dependent Depletion (CADD) scores were low, and the genes had no known function in mineral or bone metabolism; therefore these were not regarded as disease-causing variants.

#### Whole-genome sequencing

We next performed whole-genome sequencing on the 2 affected and 2 unaffected family members. In this analysis, we identified a deep intronic variant in the *PHEX* gene (NM_000444.6:c.2147 + 1197A > G) in both affected individuals but not in unaffected family members. The index was hemizygous and the mother was heterozygous for the variant. The variant is located between exons 21 and 22 (X:22264723) and this site is close to the glycosylation site, which can affect communication between cells. Exon 22 is located near the 3′-UTR regions encoding the COOH-terminal extracellular domain, with no putative zinc-binding sites or active sites. The variant was classified as likely benign and benign moderate (BP4) based on the ACMG classification guidelines, and the CADD score was 16.

**Figure 2:**
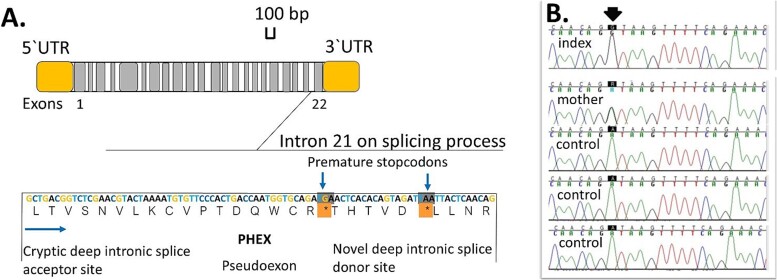
(A) The structure of *PHEX* gene according to Chandran et al.^38^ The c.2147 + 1197A > G variant will create 2 premature stop codons. (B) The hemizygous and heterozygous c.2147 + 1197A > G variant in the index and mother and the wild-type sequence in the controls confirmed by Sanger sequencing.

Based on in silico analysis, the intronic variant c.2147 + 1197A > G was predicted to cause a donor splice site gain by NetGene2 (confidence 0.83), MaxEntScan (MAXENT model increase from 2.68 to 10.86, MDD model increase from 6.90 to 15.08, MM model increase from 4.57 to 12.75, and WMM increase from 4.89 to 13.07), and SPiP (SPiPscore 0.526). In addition, SpliceAI predicted both the gain of an acceptor and donor splice site (delta PSI scores 0.26 for both, and pre-mRNA positions −84 and −1, respectively). These predictions indicated a potential pseudoexon inclusion between exons 21 and 22 (Figure 2A). Furthermore, a branch point sequence (BPS) and polypyrimidine tract (PPT) were identified (BPS and PPT score 4.10x10-03) upstream of the hypothetical pseudoexon, in addition to the canonical BPS and PTT downstream of the pseudoexon (BPS and PPT score 3.89x10-05).

Finally, the variant (NM_000444:c.2147 + 1197A > G) was confirmed by Sanger sequencing (Figure 2B).

#### The assessment and splicing predictions of the intronic PHEX variant

Based on the findings of WGS and splicing predictions of an intronic *PHEX* c.2147 + 1197A > G variant, the transcriptomic data were examined by focusing on *PHEX* transcript analyses. The analysis focused specifically on RNA samples isolated from the fibroblasts. We decided to use cultured fibroblast samples instead of whole blood samples because of the low levels of *PHEX* expression in the blood.

**Figure 3:**
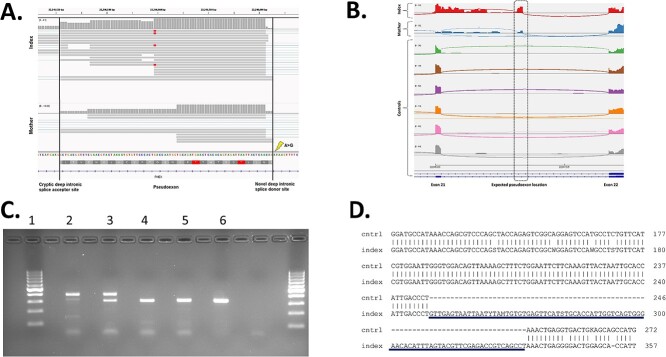
(A) IGV assessment of the predicted pseudoexon inclusion in the index and his mother. Assessing the predicted coordinates, obtained by SpliceAI, of the pseudoexon in IGV revealed read coverage in both the index and mother. The lightning bolt points to the location of the deep intronic c.2147 + 1197A > G variant, the black vertical lines indicate the predicted novel deep intronic splice sites, and the correct pseudoexon amino acid reading frame is given which indicates the presence of premature stop codons. No read coverage was detected at these genomic coordinates in the controls. (B) Alternative splicing in the index and mother causing a pseudoexon inclusion. Splicing of *PHEX* exons 21 and 22 was assessed with a sashimi plot in the index, mother, and controls. An aberrant RNA splicing pattern was detected solely in the index and mother. The hemizygous c.2147 + 1197A > G variant in the index causes full inclusion of the pseudoexon, while the heterozygous c.2147 + 1197A > G variant in the mother causes both inclusion and exclusion of the pseudoexon. The black square dotted line indicates roughly the expected location of the pseudoexon. (C) Pseudoexon on 2 % agarose gel: 1. 100 bp DNA ladder 2. Index 3. Mother 4-6. Controls. The length of the bands is 84 bp longer in the mother and index than in the controls. (D) The *PHEX* cDNA sequencing of the index and control. The sequences have been aligned using BLAST. The index has a longer sequence than the control. IGV, Integrative Genome Viewer.

Manual assessment of the intronic region between *PHEX* introns 21 and 22 revealed a potential pseudoexon inclusion around the expected genomic coordinates obtained from SpliceAI (Figure 3A). As expected, for a hemizygous variant the splicing pattern in the index supported the full inclusion of the pseudoexon containing the stop codon, while in the mother the heterozygous variant caused both the inclusion and exclusion of the pseudoexon as can be observed in the splicing pattern. The pseudoexon inclusion was not detected in any of the controls (Figure 3B). Overall, *PHEX* expression was low in the fibroblast-derived whole transcriptomic data and the identification of the pseudoexon inclusion remained uncertain.

To confirm whether this pseudoexon was indeed included, RT-PCR and Sanger sequencing were performed. Primer sequences are described in Table S1. The PCR product of *PHEX* cDNA from exons 21 to 22 was longer in patients than in controls (389 vs 306 bp). The mother, who was heterozygous for the intronic variant, showed a normal and longer band. However, the hemizygous male sample also showed a small amount of normal transcript, which appeared as a faint band on the gel (Figure 3C). Sanger sequencing of the longer PCR product confirmed the inclusion of 84 nucleotides between exons 21 and 22 (Figure 3D).

## Discussion

In this study, we describe a 2-generation family, an adult son and his mother, who had typical symptoms and clinical and biochemical findings of XLH. However, gene panels and whole exome sequencing failed to identify any pathogenic variants in the known genes related to hypophosphatemia. Therefore, we performed whole genome and whole transcriptomic data analyses of the family and discovered a deep intronic variant in *PHEX* that led to pseudoexon inclusion containing a premature stop codon in the canonical *PHEX* transcript.

The same variant as found in our study has been previously reported in a 2-generation Canadian family with inherited hypophosphatemia.^7^ However, to identify this intronic variant, we used different methods than those used in the study by Grimbly et al. who extracted RNA from urine-derived cells to screen *PHEX* cDNA. The Canadian index had hypophosphatemia, dental abscesses, bone pain, lower limb cortical thickening, and femoral bowing, which were also observed in our family. Additionally, the Canadian patient had craniosynostosis with brachycephaly and frontal bossing, which was not observed in our patients. High hemoglobin levels were not reported in the Canadian patient, unlike in our Finnish family, where symptoms also included pectus excavatum, slight parenchymal calcification of the kidneys, and stress fractures. The son was more severely affected than the mother.

The Canadian and Finnish cases show that standard genetic tools are not sufficient to diagnose deep intronic variants. The deep intronic variant (c.2147 + 1197A > G) was detected using WGS and in silico tools in the Finnish family and RNA-based diagnostics in the Canadian case. Although exome sequencing covers almost 99 % of mutations in targeted gene panels,^39^ it has limitations in detecting mutations such as low degree mosaicism, deep intronic variants, small CNVs, and mutations in repeat regions and high GC regions. In a recent study of 831 patients with XLH, 63 % of the patients were “*PHEX* positive,” 32 % were “*PHEX* negative,” and the rest of the patients had a *PHEX* variant of uncertain significance.^40^ Indeed, the authors speculate that some “*PHEX* negative” individuals may have a deep intronic *PHEX* variant underlying the disease. These deep intronic variants can only be detected by RNA-based diagnostics or WGS.

Our results indicate that the combination of WGS and in silico tools, with carefully selected filtering thresholds, can be used to identify variants predicted to impact RNA splicing, which could subsequently be evaluated using RNA based approaches. Like urine-derived cells^7^ the expression of *PHEX* in fibroblasts is less than ideal for RNA analysis. However, RNA-seq revealed a potential pseudoexon inclusion at the exact predicted genomic coordinates, and this was subsequently confirmed with RT-PCR and Sanger sequencing. Besides the 2 deep intronic splice sites, a cryptic branch point was identified upstream of the pseudoexon. While splicing is dependent on, among other things, the tissue origin^41^ our results using fibroblasts show a similar splicing pattern as in urine-derived cells^7^ thereby strengthening the hypothesis that the same aberrant splicing pattern might occur in osteocytes.

The intronic variant is likely to affect RNA splicing, however; the exact mechanism by which pseudoexons affect PHEX protein function is unknown. A premature stop codon usually affects mRNA stability and degradation, but the mechanism does not work when the codon is located near the 3’ end of an exon-exon junction. Grimbly et al. speculated that 33 amino acids at the C-terminus would be replaced by 17 amino acids, and this change would eliminate 2 cysteine residues from the disulfide bridges of the PHEX protein, which would have a detrimental effect on the 3D configuration of the PHEX protein.^7^ This intronic variant can probably be considered pathogenic, as other pathogenic variants are known to be in the exon 22 region that eliminate amino acids at the C-terminus, such as the *PHEX* variant c.2239C > T (p.Arg747Ter),^5^ which results in the loss of 3 amino acids at the C-terminus. Changes in the C-terminal region affect the ability of PHEX to process substrates.

However, the effects of the intronic variant on PHEX remain unclear. Unfortunately, the transcriptomic data reads were insufficient, and the expression levels between patients and controls could not be determined in our study. Overall, deep intronic variants are difficult to detect using current standard methods, as only 2 deep intronic *PHEX* variants have been detected in past studies.^6^^,^^7^ These current methods also cause a long delay in diagnosis and getting the right treatment.

We propose that this intronic variant, now confirmed in 2 independent studies, should be added to the gene panels used in the diagnostics of hypophosphatemia. Our study also suggests that WGS could be useful in cases with a negative gene panel. Grimbly et al. discovered the variant using RNA-based diagnostics,^7^ which, if developed into a method more suitable for clinics, could be one solution to improve the detection of deep intronic variants in the future. In summary, in individuals with the typical clinical features of XLH and negative results from the gene panel and WES, the disease may be caused by a deep intronic *PHEX* variant that can be detected by WGS and confirmed by RT-PCR and Sanger sequencing. Our findings highlight the importance of deep-intronic *PHEX* variants in the diagnosis of XLH. These methods can be useful for investigating other rare diseases for which genetic causes have not been identified, and WES analyses have not revealed any potential pathogenic variants.

## Supplementary Material

Supplements_ziae169

## Data Availability

The data analyzed for the study are not publicly available due to patients’ privacy. The data of this study are available from the corresponding author upon reasonable request.
