# Supplementary material for: A deep intronic PHEX variant associated with X-linked hypophosphatemia in a Finnish family
Source: JBMR Plus. 2024 Dec 23;9(2):ziae169. doi: 10.1093/jbmrpl/ziae169 (PMC11772523; doi:10.1093/jbmrpl/ziae169)
Supplement: Supplements_ziae169 [file supplements_ziae169.pdf]

A deep intronic *PHEX* variant associated with X-linked hypophosphatemia in a Finnish family

Laura Koponen[1,2], Minna Pekkinen[1,2,3], Jelmer Legebeke[4], Mari Muurinen[1,2,3], Salla Rusanen[2], Shabir Hussain[1], Fan Wang[4], Pasi I. Nevalainen[5], Outi Mäkitie[1,2,3,4,6]

[1.] Research Program for Clinical and Molecular Metabolism, Faculty of Medicine, University of Helsinki, 00014 Helsinki, Finland

[2.] Folkhälsan Research Center, 00290 Helsinki, Finland

[3.] Children's Hospital, University of Helsinki and Helsinki University Hospital, 00014 Helsinki, Finland

[4.] Department of Molecular Medicine and Surgery, Karolinska Institutet, Stockholm, Sweden

[5.] Rare Diseases Unit and Endocrine Unit, Department of Internal Medicine, Tampere University Hospital, and Faculty of Medicine and Health Technology, Tampere University, Tampere, Finland

[6.] Clinical Genetics, Karolinska University Hospital, Stockholm, Sweden

ORCiD numbers: *0000-0001-8971-4306 (L. Koponen); 0000-0003-2947-4683 (M. Pekkinen); 0000-0003-1194-8959 (J. Legebeke); 0000-0002-9197-1288 (S. Rusanen); 0000-0001-5744-4292 (S. Hussain); 0000-0003-4888-8793 (F. Wang); 0000-0002-7397-6122 (PI Nevalainen); 0000-0002-4547-001X (O. Mäkitie)*

Corresponding author: Outi Mäkitie, Folkhälsan Research Center, P.O.Box 63, FIN-00014 University of Helsinki, Helsinki, FINLAND Phone: +358-9-191 25453, Fax. +358-9-191 25073 Email: outi.makitie@helsinki.fi

Keywords: XLH, genetic skeletal disorders, splicing, Next Generation sequencing (NGS), whole genome sequencing, transcriptomics

Supplemental Figure 1. Whole genome sequencing data analysis in four family members (two affected and two unaffected individuals). The gene list included genes previously linked to dominant or recessive disorders with low phosphate levels: *ALPL*, *NRAS*, *PTH1R*, *CASR*, *SLC34A1*, *FGFR1*, *HRAS*, *GFG23*, *KRAS*, *GATM*, *NF1*, *NHERF1*, *AP2S1*, *GNAS*, *POLRMT*, *ABCC6*, *PHEX*, *HMGCS2*, *SLC5A6*, *SLC2A2*, *DMP1*, *ENPP1*, *FAM20C*, *NDUFAF6*, *DGAT1*, *ALDOB*, *SLC34A3*, *CYP2R1*, *INPPL1*, *VDR*, *CYP27B1*, *FAH*, *CTNS*, *CLCLN5*, *SASH3*, *POLRMT*, and *ABCC6*.

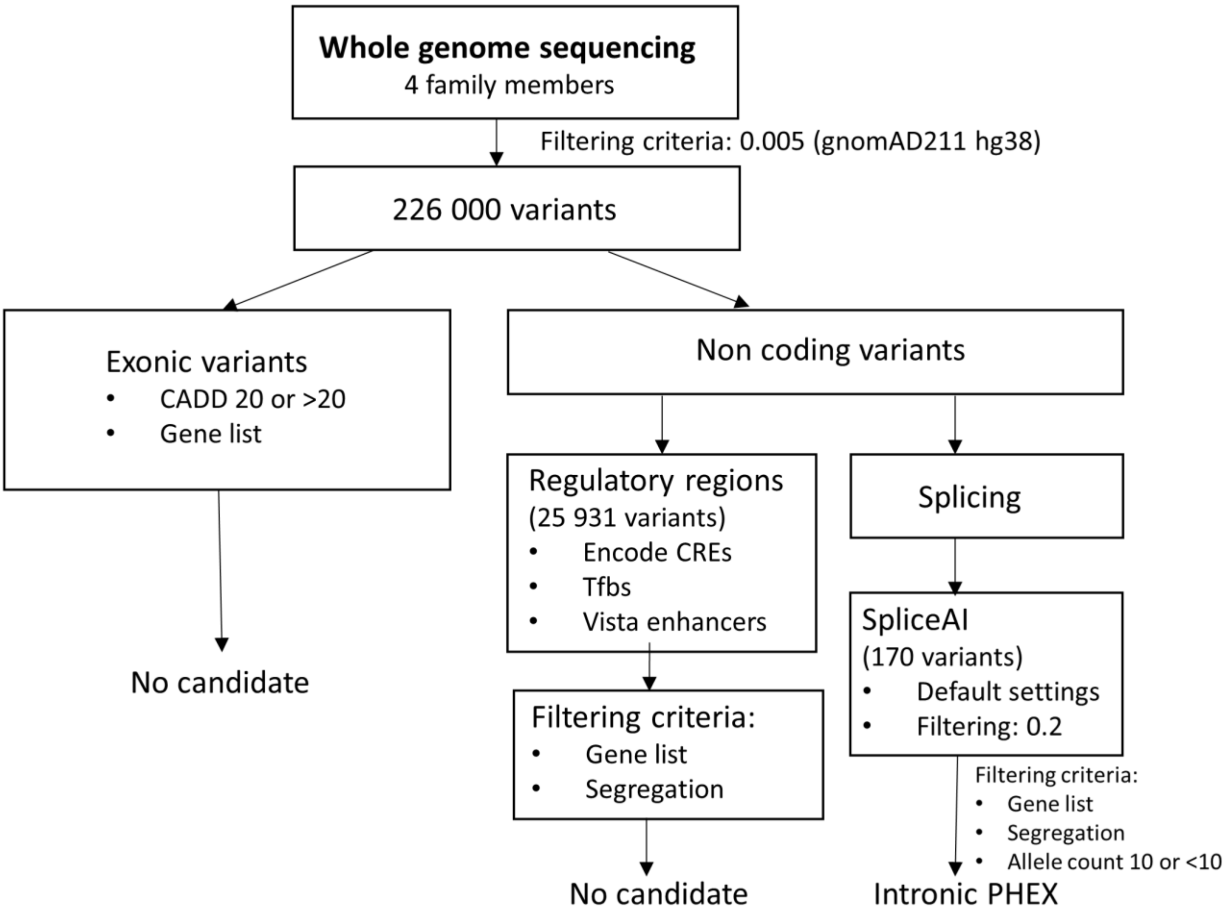

CREs, cis-regulatory elements; TFBS, transcription factor binding sites

Supplementary Table 1. Oligo names and primer sequences concerning RT-PCR and Sanger sequencing.

**Oligo Name**	**Sequence**
cPHEX_ex1_ex2_F	TCTACGGCCCTTCTGATGGA
cPHEX_ex1_ex2_R	GATGCATTCTGGCTTCAGGC
cPHEX_ex1_ex2_F	TCTACGGCCCTTCTGATGGA
cPHEX_ex1_ex2_R	GATGCATTCTGGCTTCAGGC
cPHEX_ex20_ex21_F	ACCAGGCATCACATTCACCA
cPHEX_ex20_ex21_R	TTTGGACTTGTTCTCGGGCA
cPHEX_ex20_ex21_F	ACCAGGCATCACATTCACCAA
cPHEX_ex20_ex21_R	TTGGACTTGTTCTCGGGCAG
cPHEX_ex21_ex22_F	CTACAGACCAGAAGCTGCCC
cPHEX_ex21_ex22_R	CAAGCAGGTCACCTACCCAA
